# Computer order entry systems in the emergency department significantly reduce the time to medication delivery for high acuity patients

**DOI:** 10.1186/1865-1380-6-20

**Published:** 2013-07-05

**Authors:** Shahbaz Syed, Dongmei Wang, Debbie Goulard, Tom Rich, Grant Innes, Eddy Lang

**Affiliations:** 1 Faculty of Medicine, University of Calgary, 3330 Hospital Dr. NW, Calgary, Alberta T2N 4M1, Canada

## Abstract

**Background:**

Computerized physician order entry (CPOE) systems are designed to increase safety and improve quality of care; however, their impact on efficiency in the ED has not yet been validated. This study examined the impact of CPOE on process times for medication delivery, laboratory utilization and diagnostic imaging in the early, late and control phases of a regional ED-CPOE implementation.

**Methods:**

Setting: Three tertiary care hospitals serving a population in excess of 1 million inhabitants that initiated the same CPOE system during the same 3-week time window. Patients were stratified into three groupings: Control, Early CPOE and Late CPOE (*n* = 200 patients per group/hospital site). Eligible patients consisted of a stratified (40% CTAS 2 and 60% CTAS 3) random sample of all patients seen 30 days preceding CPOE implementation (Control), 30 days immediately after CPOE implementation (Early CPOE) and 5–6 months after CPOE implementation (Late CPOE). Primary outcomes were time to (TT) from physician assignment (MD-sign) up to MD-order completion. An ANOVA and *t*-test were employed for statistical analysis.

**Results:**

In comparison with control, TT 1st MD-Ordered Medication decreased in both the Early and Late CPOE groups (102.6 min control, 62.8 Early and 65.7 late, *p* < 0.001). TT 1st MD-ordered laboratory results increased in both the Early and Late CPOE groups compared to Control (76.4, 85.3 and 73.8 min, respectively, *p* < 0.001). TT 1st X-Ray also significantly increased in both the Early and Late CPOE groups (80.4, 84.8 min, respectively, compared to 68.1, *p* < 0.001). Given that CT and ultrasound imaging inherently takes increased time, these imaging studies were not included, and only X-ray was examined. There was no statistical difference found between TT discharge and consult request.

**Conclusions:**

Regional implementation of CPOE afforded important efficiencies in time to medication delivery for high acuity ED patients. Increased times observed for laboratory and radiology results may reflect system issues outside of the emergency department and as a result of potential confounding may not be a reflection of CPOE impact.

## Background

Calgary, Alberta has recently become the first Canadian city to implement a regional CPOE (computer physician order entry) system at each of its hospital’s emergency departments (known as Sunrise Clinical Manager, or SCM). CPOE systems have been successfully implemented among inpatient services, but their efficacy in the ED has yet to be validated. Patients in the ED are often critically ill and require timely intervention. It is therefore essential to design time-saving techniques to maximize efficiency and test turnaround time. One of the greatest advantages of CPOE systems is their ability to enhance the delivery of safe health care in a relatively chaotic environment. It is estimated that around 44,000-98,000 inpatient Americans die each year because of avoidable medical errors [1] and that a hospital patient is exposed to an average of one medication error per day [2]. Canadian studies examining safety suggest that hospital error occurs in approximately 7.5 per 100 hospital admissions; preventable events occurred in 37% of these patients and death in 20% [3]. Studies examining safety after the implementation of CPOE suggest that medication delivery error can be minimized by up to 80% and that complications resulting in serious morbidity or mortality can be decreased by 55% [4]. A direct analysis demonstrated that medication delivery through a CPOE system decreased the likelihood of a medication error by 48%; with an extrapolation to American populations this would correlate to approximately 17.4 million fewer medication errors per year [5]. Secondary to this, CPOE is designed to decrease delays related to order completion and processing, allows for point-of-care order entry, provides error checking to minimize repeat dosing or allergic reactions, and allows for easy access to previous medical records [6]. A direct correlation between test turnaround time and emergency department wait times has been demonstrated previously [7]. To this end, CPOE offers a potential mechanism targeted towards better patient safety specific goals, allowing for real-time patient identification, drug allergies, adverse reactions and treatment conflicts. The system also allows for portability, as well as patient confidentiality.

Potential pitfalls of CPOE are related to slower order entry and diminished response time in an emergency setting where urgent data are critical. Automation may also create a false sense of security in regards to error checking, and alert fatigue may result from the announcement of erroneous errors [8]. These factors are attributed to an increased mortality rate following the implementation of CPOE in a Pittsburgh pediatric ICU. Recent criticism of this work, however, questions whether the increased mortality was secondary to the actual usage of CPOE, or whether the manner in which the hospital implemented CPOE was a confounding factor [8]. Further research needs to be done to evaluate these potential negatives of CPOE.

The importance of providing efficient and optimized health care cannot be overlooked, especially as emergency department wait times continue to increase. Therefore, if CPOE is effectively able to minimize time-consuming processes, wait times should likewise decrease. In this study, we sought to examine our regional implementation of CPOE to provide a comprehensive evaluation of both the pros and cons of the system in an emergency setting. This study in particular examined the impact of CPOE on process times for medication delivery, laboratory utilization and imaging in the early, late and control phases of a regional ED-CPOE implementation.

## Methods

### Setting

Results were obtained from three Calgary hospitals in a retrospective fashion via chart review or computer data analysis.

### Design

This study was designed to be a retrospective before-and-after analysis, examining time to (TT) medication delivery, laboratory and imaging completion for patients within the ED. To be included, the patient needed to have received all of preceding services in one ED visit with times appropriately documented. The control group consisted of a randomly selected but representative population size of 200 patients/hospital site seen in the 30 days prior to CPOE implementation who were given medication, with laboratory and imaging orders, stratified by the time and day of presentation. The early CPOE group consisted of 200 patients/site, as a randomized but representative population of patients who had medication, laboratory and imaging orders in the 30 days following the implementation of CPOE stratified by the time and day of presentation. The late CPOE group consisted of a population group of 200 patients/site randomized, but representative of all patients seen in a 5–6-month period following CPOE implementation, who had medication, laboratory test and imaging orders stratified by the time and day of presentation. These patients were selected by compiling a list of patients who visited a particular site hospital during the study time frame and using a random number generator to select the study group. Each of these groups was stratified into 40 CTAS 2 and 60 CTAS 3 patients. CTAS (Canadian Triage and Acuity scale) level 2 dictates that patients need to be seen by a physician within 15 min 95% of the time, while CTAS 3 patients need to be seen by a physician within 30 min 90% of the time. In all three groups exclusion criteria were applied if patients were admitted from the ED but had no consultation request time, no physician ordered laboratory tests, imaging or medications, medications given by RN protocol or verbal order, or if they were initially triaged to fast track (patients for whom preexisting nursing protocols are initiated or are seen by physicians briefly for fast-tracked orders), minor treatment or resuscitation room.

### Outcomes

Primary outcome measurements were time from MD sign-up to (1) first physician ordered medication administration, (2) first physician ordered test result and (3) first imaging performed. Secondary outcomes included time for MD signup to disposition.

### Analysis

The TT 1st MD-ordered medication, laboratory test result and X-ray were compared using an ANOVA analysis to evaluate statistical differences between groups in each of the sample groups. Each group was compared in regards to TT discharge and TT consult request via an ANOVA to detect for statistical significance.

## Results

During the study periods a total of 1,800 patient records were examined, 600 within each study group (200 patients/per site/per study group). There was no significant difference among baseline patient characteristics (Table 1). There was also no significant difference in mean daily census, but there was a difference in mean time to MD among CTAS 2 and 3 patients during the early phase of CPOE implementation compared to the control and late CPOE groups. There was also an increase in the ED length of stay during the early CPOE phase in comparison to control and late CPOE groups (Table 1).

**Table 1:** Baseline characteristics for patients and study period

**Patient**	**Control (*n *= 600)**	**Early CPOE (*n *= 600)**	**Late CPOE (*n *= 600)**
**Age**	57.2	57.5	58.0
**Gender (male)**	283 (47.2%)	320 (53.3%)	292 (48.7%)
**EMS arrival: *n *(%)**	254 (42.3%)	279 (46.5%)	262 (43.7%)
**Triage time (D/Eve/N)**			
Day (07–14:59)	256 (42.7%)	220 (36.7%)	246 (41.7%)
Evening (15–22:59)	238 (39.7%)	275 (45.8%)	265 (44.2%)
Night (23:00–06:59)	106 (17.7%)	105 (17.5%)	89 (14.8%)
**First location after triage**			
ED stretcher	130 (21.7%)	124 (20.7%)	140 (23.3%)
RAZ (rapid assessment zone)	8 (1.3%)	6 (1.0%)	12 (2.0%)
Intake area	11 (1.8%)	19 (3.2%)	8 (1.3%)
Waiting room	451 (75.2%)	451 (75.2%)	440 (73.3%)
**Period characteristics**			
Mean daily census*	577	590	569
Mean time to MD (min)*	122	145	113
Mean ED LOS admitted patients (h)**	17.44	18.45	16.45

*CTAS 2 and3 in main ED, **LOS (length of stay) from triage to disposition.

In comparison to the control group, there was a statistically significant decrease in the time to MD-ordered medication delivery for both the early and late CPOE groups, from 102.6 min to 62.8 and 65.7 min for early and late CPOE, respectively (*p* < 0.001).

**Figure 1:**
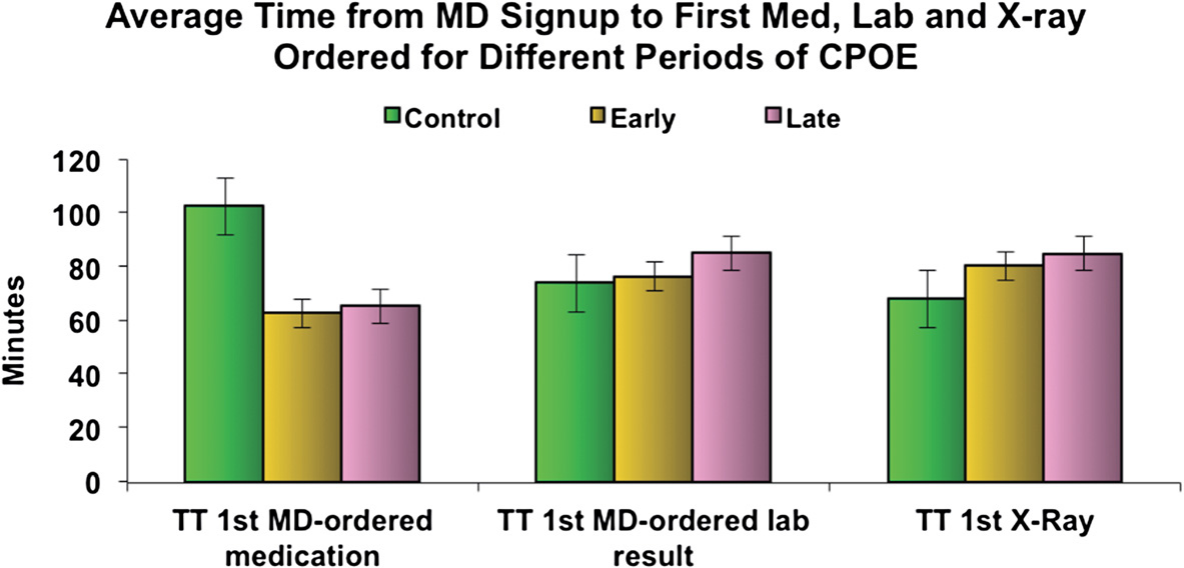
Average time from MD signup to first med, lab and X-Ray ordered for different periods of CPOE.

The time to MD-ordered laboratory test results was significantly increased in both the early and late CPOE groups: 76.4 and 85.3 min, respectively, compared to 73.8 min in the control group (*p* < 0.001) (Figure 1).

Time to MD-ordered X-ray was also significantly increased in both early and late CPOE groups: 80.4 and 84.8 min, respectively, in comparison to 68.1 min in the control group (*p* < 0.001) (Figure 1).

There was no significant difference between the groups in terms of time to discharge for control, early or late CPOE groups: 5.72, 5.42 and 6.01 h, respectively (*p* > 0.05).

**Figure 2:**
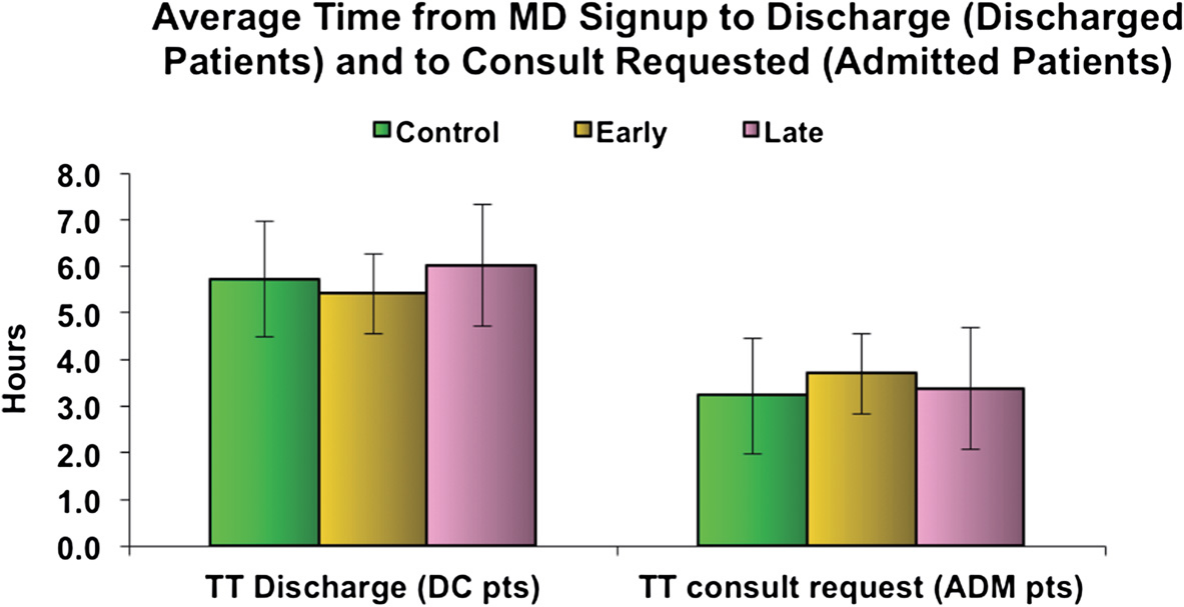
Average time from MD signup to discharge (discharged patients) and to consult requested (admitted patients).

There was also no significant difference for time to consult request among admitted patients among control, early or late CPOE groups: 3.24, 3.69 and 3.39 h, respectively (*p* > 0.05) (Figure 2).

## Discussion

In this study, we attempted to examine the overall impact of CPOE on factors that might be essential for minimizing overall wait room times. Studies have shown a direct correlation between test turnaround time and patient wait time in the ED. We found that the implementation of computer entry systems decreased the time to medication delivery by greater than 30 min at all CPOE time points. Contrary to our hypothesis, however, we found that time to laboratory studies and first imaging increased slightly in the CPOE era. For imaging or blood work to be completed prior to CPOE, an order would be written on the chart and then the nurse would enter the orders in the electronic ordering system on behalf of the physician. For diagnostic imaging, the nurse would call for a porter to take the patient to imaging. For laboratory ordering, the nurse would draw the blood work and then send the blood samples to the laboratory via a tube system. Currently, with CPOE, the order for task completion goes straight to the requested department, bypassing the nursing step of entering the tests in the electronic system. For laboratory orders, the nurse still has to draw the blood samples to send to the laboratory. Intuitively, we predicted that this would result in decreased times to imaging, medication and blood work completion. A study by *Stelle* and colleagues demonstrated a significant difference in time to medication delivery and radiology completion following the implementation of CPOE in a Denver hospital [9]. This work focused on the test turnaround time on both medical and surgical wards following a CPOE implementation. It remains to be seen whether or not this analysis translates well to emergency department care. For example, the study demonstrates a pre-CPOE time to radiology of 1,860 min and a post-implementation time of 714 min. While this reduction in time to test delivery is drastic, imaging in the ED occurs at a much more rapid pace (with imaging occurring in under 85 min in all of our groups in this study). Further research done by Mekhjian et al. demonstrated a similar decrease in time to medication, laboratory and radiology completion following CPOE implementation [10]. Once again, however, this study focused on inpatients, and its ability to be translated to emergency department care has yet to be proven. A recent systematic review was performed by Georgiou and colleagues examining the effect of CPOE on work processes in emergency departments. The group’s analysis found 22 studies meeting their inclusion criteria, but no studies that measured support systems and their effect on patient flow or clinical work. The analysis identified increased safety profiles associated with CPOE and increased time spent on computers, but nothing regarding flow optimization in the emergency department [11].

There is currently no literature looking at medication delivery, laboratory and imaging completion in the ED, but evidence on CPOE systems suggests that these processes should be completed faster. It is possible that our increased times to laboratory and radiology results are secondary to processes external to the ED or the processing of information on the receiving end of CPOE (i.e., radiology or laboratory services). More work needs to be done to test this postulate, but it is possible that the increased time to testing is related to processes outside of the ED’s control. Many new systems have been implemented to help minimize wait room times (such as fast track and intake areas in the ER), and it is possible that these increased services have also resulted in earlier test ordering on patients, thus backing up radiology and laboratory services even further. Given that there is no literature on this topic, it is clear that more research needs to be done to investigate this finding in our research.

One of the biggest physician complaints about CPOE is time spent away from patients, minimized nursing interaction, increased time spent at computers and decreased productivity. A recent study illustrated that physician time with patients was not decreased following the implementation of CPOE; however, there was significantly decreased time spent discussing patient care with nurses [12]. Likewise, there was increased time spent at the computer, but the majority of this time was not spent inputting orders, but rather reviewing patient medical files and previous visits – resulting in increased time spent on indirect patient care [12], allowing emergency physicians to compile accurate information in a more timely fashion.

Ongoing studies examining the efficacy of CPOE are critical, but they are limited by the inevitable multitude of factors affecting productivity in the emergency department, including external processes as well as those within in the ER.

## Conclusions

As we move forward in modern medicine, we are simultaneously engaging in advancing technologies as well. The use of computer systems in the emergency department will soon become a universal reality. The implementation of CPOE at a regional level in Calgary has resulted in decreased time to medication delivery. We noted increased times for laboratory and imaging requests, which may be secondary to processes external to the ED, but more research in this regard is required.

## Competing interest

None of the authors have any financial or other conflicts to disclose.

## Authors’ contributions

The entire project was a collaborative effort, with DG, GI, TR and EL helping in the planning and design for the study. SS and DW were responsible for data collection and analysis, with DW conducting statistical analysis. SS prepared, edited, distributed and published the manuscript, with all authors reading and approving the final manuscript.
